# Elevated 14,15- epoxyeicosatrienoic acid by increasing of cytochrome P450 2C8, 2C9 and 2J2 and decreasing of soluble epoxide hydrolase associated with aggressiveness of human breast cancer

**DOI:** 10.1186/1471-2407-14-841

**Published:** 2014-11-18

**Authors:** Xiaolong Wei, Donghong Zhang, Xiaowei Dou, Na Niu, Wenhe Huang, Jingwen Bai, Guojun Zhang

**Affiliations:** Department of Pathology, Cancer Hospital of Shantou University Medical College, Shantou, 515031 Guangdong China; Department of Clinical Laboratory, Peking Union Medical College Hospital, Beijing, 100730 China; The Breast Center, Cancer Hospital of Shantou University Medical College, Shantou, 515031 Guangdong China; Department of Pathology, Weifang Medical University, Weifang, 261042 Shandong China

**Keywords:** Soluble epoxide hydrolase, Cytochrome P450, Breast cancer, Proliferation, Migration

## Abstract

**Background:**

Epoxyeicosatrienoic acids (EETs) are derived from arachidonic acid by cytochrome P450 (CYP) and metabolized by soluble epoxide hydrolase (sEH). EETs have been associated with cardiovascular disease, diabetes and several cancer diseases. However, the distribution in tissue and role of CYP2C8, 2C9, 2J2 and sEH in human breast carcinogenesis remains uncertain.

**Methods:**

Breast cancer (BC) and adjacent noncancerous tissue was obtained from 40 breast cancer patients in the Chaoshan region in China from 2010 to 2012. The level of 14,15-EET/14,15-DHET in BC patients was detected by ELISA; the expression and distribution of CYP2C8, 2C9, 2J2 and sEH was determined by quantitative RT-PCR and immunohistochemical staining; and cell proliferation and migration was analyzed by MTT and transwell assays, respectively.

**Results:**

The median 14,15-EET and 14,15-EET/DHET level was 2.5-fold higher in BC than noncancerous tissue. The mRNA and protein levels of CYP2C8, 2C9 and 2J2 were higher, and sEH was lower in BC than noncancerous tissue. Furthermore, CYP2C8 and 2C9 protein levels positively correlated with Ki67 status, and CYP2J2 levels positively correlated with histological grade and tumor size. The sEH protein level negatively correlated with tumor size, estrogen receptors and Ki67. In MDA-MB-231 cells, siRNA knockdown of CYP2C8, 2C9 or 2J2 reduced cell proliferation, by 24.5%, 29.13%, or 22.7% and decreased cell migration by 49.1%, 44.9%, and 50.9%, respectively. Similarly, with adenovirus overexpression of sEH, both cell proliferation and migration rates were reduced by 31.4% and 45.8%, respectively.

**Conclusions:**

The present study shows that elevated EET levels in BC tissues are associated with upregulation of CYP2C8, 2C9, and 2J2, and downregulation of sEH, and are also associated with aggressive cell behavior in BC patients.

## Background

Breast cancer (BC) is the most frequently diagnosed cancer and the leading cause of cancer deaths in women in both developed and developing countries worldwide. In 2008, 1.38 million new cases of BC were diagnosed and 458,400 people died due to BC [1]. The etiology of BC appears to be related to a long menstrual history, nulliparity, recent use of postmenopausal hormone therapy or oral contraceptives, late age at first birth and alcohol consumption [2]. However, substantial numbers of patients still experience metastatic disease, and further improvements in survival depend on a better understanding and identification of cellular targets within the malignant cell for novel therapeutic development and for targeting of optimal therapies. Thus, the exact causes of BC and its malignant potential are still unclear.

Epoxyeicosatrienoic acids (EETs), derived from arachidonic acid by cytochrome P450 (CYP), promote the pathogenesis of various human cancers [3–7]. Four regioisomeric EETs (5,6-EET, 8,9-EET, 11,12-EET, and 14,15-EET) are active lipid signaling molecules and are anti-inflammatory, proliferative, and angiogenic, and easily spread within several tissues under both physiologic and pathologic conditions. In humans, CYP2C8, 2C9 and 2J2 subfamily members participate in the synthesis of EETs, which are then quickly metabolized by soluble epoxide hydrolase (sEH) into their respective diols in most tissues [8, 9]. Thus, the balance of CYP2C8, 2C9, and 2J2, as well as sEH expression is responsible for sustaining EET concentration.

CYP2C8, 2C9, 2J2 and sEH expression has been detected in several tumor tissues and cells, which supports a role for EETs in cancer. Both CYP2C8 and 2C9 are highly expressed in human malignant neoplasms, such as renal carcinoma, lung adenocarcinoma (but not lung squamous cell carcinoma), ductal breast carcinoma, colon adenocarcinoma, basal cell carcinoma, bladder transitional cell carcinoma, ovarian adenocarcinoma, endometrial carcinoma, and prostate adenocarcinoma. In contrast, CYP2C8 expression has been found to be downregulated five-fold in esophageal adenocarcinoma as compared with para-cancerous tissue [10]. CYP2J2 expression is elevated in human malignant tumors, such as esophageal, liver, breast, lung, and colorectal cancers, and high levels of EETs are detected in urine and blood of patients with these cancers [3, 11]. In contrast, pancreatic or prostate adenocarcinoma or BC tissue do not show CYP2J2 expression, and the enzyme is detected in less than 50% of lung squamous cell carcinoma and less than 15% of lung adenocarcinoma samples [10, 12, 13]. This decrease in arachidonic acid epoxidation in certain tumors may allow arachidonic acid to be metabolized to other eicosanoids [12]. Loss of sEH has been reported in renal tumors, hepatocellular carcinoma and hepatoma cells [10, 14, 15], which would result in an enhanced role of EETs in carcinogenesis. However, upregulation of sEH expression has been found in other types of cancers, such as seminoma, cholangiocarcinoma, and advanced ovarian cancer, as compared with normal tissue or early-stage cancer [3, 10, 11].

Although many studies have focused on EET synthesis and metabolic enzymes in several cancers, the characteristics and roles of EET isoforms such as CYP2C8, 2C9, and 2J2, and sEH in BC remains poorly understood. A recent microarray assay of BC tissue showed CYP2C expression in 80% of the tissues, with weak or moderate immunoreactivity [13], whereas other studies found high prevalence and modest or strong immunoreactivity [3, 11]. We conducted a retrospective investigation of the level of EET and expression and distribution of CYP2C8, 2C9, and 2J2 and sEH in human BC tissue and adjacent noncancerous tissue. We further investigated the role of EETs and sEH during breast cancer proliferation and migration.

## Methods

### Patients

The study protocol was performed according to the Declaration of Helsinki and was approved by the Ethics Committee of the Cancer Hospital of Shantou University Medical College. All patients were from the Shantou region of China and gave their signed informed consent for the use of biological samples.

All patients underwent modified radical mastectomy or mastectomy, and no patients received lumpectomy. Fresh noncancerous tissues were collected at least 5 cm away from the margins of tumors for paired malignant lesions from 40 patients (mean age 44.5 ± 8.7 years) in the Cancer Hospital of Shantou University Medical College from 2010 to 2012. Non-cancerous tissue, confirmed by a pathologist, was defined as normal breast tissue not presented with ductal carcinoma in situ (DCIS), atypical hyperplasia or benign breast disease. Changes in tumor size, node metastasis and histological grade were determined according to the World Health Organization histological classification criteria [16].

### 14,15-EET/DHET detection

Episomal and esterified 14,15-DHET (including episomal and transformed DHET from 14,15-EET by sEH) in BC tissues was determined by use of an ELISA kit (Detroit R&D, USA). Briefly, we homogenized 30 mg tissue in 0.4 mL of H_2_O, containing 0.001 mg TPP (triphenylphosphine, an antioxidant), for all tumor and non-tumor tissues before ethyl acetate extraction. Total protein concentration was measured with a BSA kit according to the protocol (Takara Biotechnology [DALIAN] Co.) and used to normalize EET and DHET measurements. Ethyl acetate extracts were incubated in ethanol and acetic acid for 18 h at room temperature to allow complete EET hydrolysis to DHET. Then, 130 uL of Sample Dilution Buffer was added to make a stock sample solution. The final pH was adjusted to pH 7.4, if necessary, then DHET, which included DHET converted from EET, was measured using a 14,15-DHET ELISA kit. At the same time, the DHET level was measured without hydrolysis of EET in the same sample, then subtracted from the EET + DHET level to obtain the EET level in the sample. Inability to detect levels of EETs indicated that EET was totally hydrolyzed to DHET. The efficiency of conversion of EET to DHET according to the free of EET formation activity measurement after the EET hydrolyzed to DHET by ethanol and acetic acid.

### Immunohistochemical (IHC) staining

After excision, clinical samples were fixed immediately in 4% paraformaldehyde for 24 hr, embedded in paraffin and sectioned at 4 μm thickness for IHC staining with primary antibody for CYP2C8 (1:100, Proteintech Group, Chicago, IL, USA), 2C9 (1:100, Biosynthesis, Beijing), and 2J2 (1:100, Abgent, San Diego, CA), as well as for sEH (1:100, Santa Cruz Biotechnology, Santa Cruz, CA) as described [13, 17]. IgG or phosphate-buffered saline (PBS) was a negative or blank control, respectively. Then, the slides were incubated with polyclonal peroxidase-conjugated anti-mouse/rabbit IgG (PV9000; Zymed Laboratories, South San Francisco, CA). Sections were stained with 100 μl AEC chromogen (Maxim.bio Co.) and restained with haematoxylin for visualization of nuclei.

The scoring of positive immunoreactivity was as described previously [4, 18] with modifications: 0, <25%; 1, 25–50%; 2, 50–75%; 3, >75%. The intensity of staining was scored as 0, absence of signal; 2, low-intensity signal (light red); 2, moderate-intensity signal (red); and 3, high-intensity signal (dark red). The final score for each case was the total of the frequency and intensity scores, with the following classification: 0 or 1, negative (−); 2 or 3, moderately positive (+); and 4 to 6, highly positive (++).

### Cell culture, transfection and infection

Human BC MDA-MB-231 cells were maintained in DMEM (Invitrogen, Carlsbad, CA) supplemented with 10% heat-inactivated fetal bovine serum at 37°C in a humidified atmosphere containing 5% CO_2_. After culture for 24 hr at 50% to 60% density, cells were transfected with 40 μmol/L siRNA pools for CYP2C8, 2C9 or 2J2 by the Jet PEI method (Polyplus, San Marcos, CA) or infected with adenovirus sEH (Ad-sEH) [19], a recombinant Ad expressing human-EPXH2. Ad-GFP was an infection control.

### Quantitative reverse transcription-PCR (qRT-PCR)

Total RNA was isolated from tissue and cells with use of TRIzol reagent (Invitrogen), and 0.5 μg RNA was converted to cDNA with the SuperScript II Reverse Transcriptase kit (Invitrogen). Primers for CYP2C8, 2C9, 2J2, sEH and β-actin were as we described previously [17]. qRT-PCR amplification involved the PrimeScript Real-Time RT-PCR reagent kit (Takara Biotechnology [DALIAN] Co.) and Applied Biosystems Prism 7300 (Invitrogen). DNase-treated RNA was amplified without reverse transcriptase as a negative control. Human hepatocellular carcinoma tissue RNA was a positive control and water was a blank control. Amplification of β-actin was an internal control. The relative expressions of CYPs and sEH were normalized to their corresponding normal control tissue.

### MTT assay

MDA-MB-231 cells were inoculated at 5000 cells per well of a 96-well plate, allowed to attach for 24 hr, and then treated with siRNA or Ad-sEH/Ad-GFP at the indicated amounts for another 24 hr. Cell proliferation was analyzed by use of the MTT Cell Proliferation and Cytotoxicity Assay Kit (Beyotime, China). The corrected absorbance of each sample was calculated by comparison with that of the siRNA control or Ad-GFP as an infection control.

### Cell transwell assay for migration

MDA-MB-231 cells treated with siRNA or Ad-sEH/Ad-GFP were inoculated at 2 × 10^5^ in transwell inserts with 0.8 μm pore size (Corning, New York, USA) in 24-well plates for 24 h. Cells in the bottom inside of the membranes were removed. Migrating cells on the outside membrane were washed and stained with crystal violet for 10 min. The number of migrating cells was measured by counting 5 randomly chosen fields under a microscope [20].

### Statistical analysis

Statistical analysis involved use of SPSS 16.0 (SPSS Inc., Chicago, IL). The normality of variables was assessed. The Mann–Whitney two-sample test was used to assess differences in 14,15-EET levels and mRNA expression of CYP2C8, 2C9, and 2J2, as well as sEH in BC and adjacent noncancerous tissue. Spearman correlation was used to analyze the correlation of clinicopathological variables and CYP2C8, 2C9, and 2J2, as well as sEH protein expression. Student’s t test was used for statistical analysis of cell proliferation and migration assays. Data are expressed as median (interquartile range [IQR]) or mean ± SD. Each experiment was performed at least in triplicate. P <0.05 was considered statistically significant.

## Results

### 14,15-EET levels in BC and noncancerous human tissue

**Figure 1:**
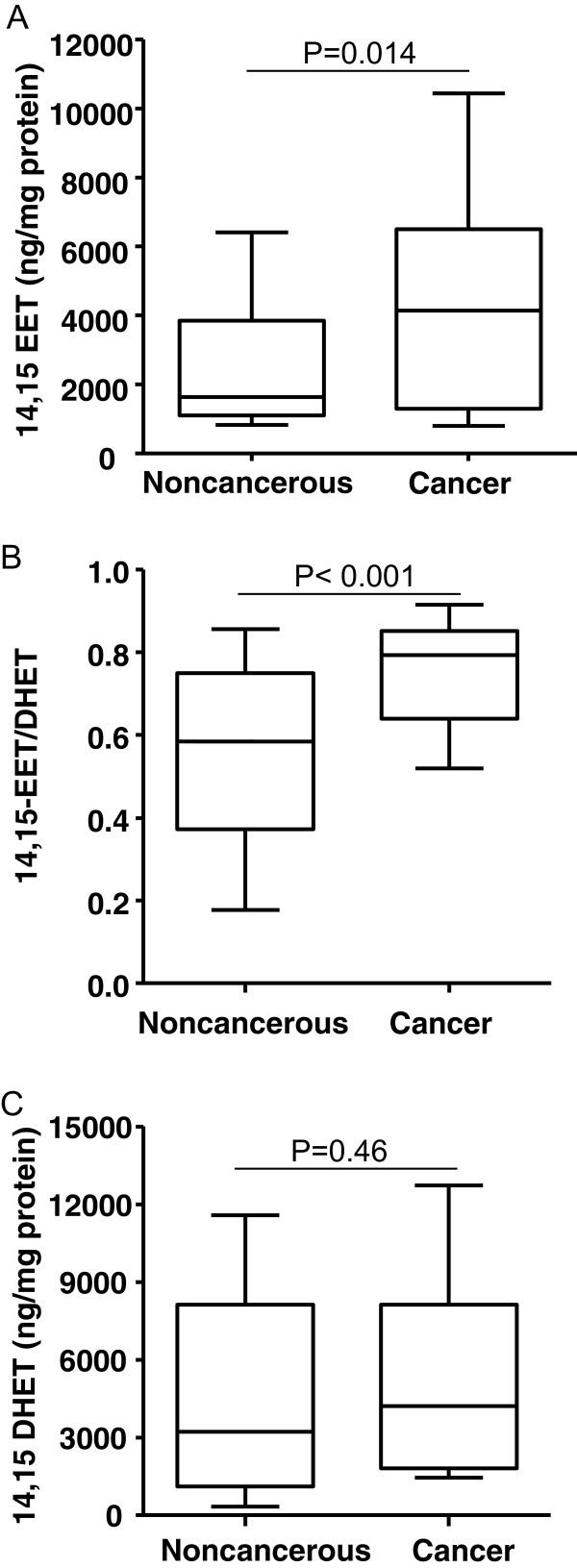
**Intracellular levels of 14,15-EET (A), 14,15-EET/DHET (B) and total 14,15-DHET (C) in breast cancer and paired adjacent noncancerous regions.** Boxes and whiskers represent the 25th–75th and 10th–90th percentiles, respectively; the median is the central line in each box. The P value was calculated by the Mann–Whitney two-sample test.

EETs and their synthetic and metabolic enzymes (CYP2C8, 2C9, 2J2 and sEH) promote angiogenesis, inflammation, and carcinogenesis [5, 6, 17]. We detected the 14,15-EET level in cancer tissue from 40 BC patients. The median 14,15-EET level was 2.5-fold higher in BC tissue than adjacent noncancerous tissue (4145.9 [IQR 1299.8-6500.0] vs. 1634.4 [1092.5-3844.7] ng/mg protein; p =0.01) (Figure 1A) and the median ratio of 14,15-EET to episomal 14,15-DHET was higher in BC tissue than noncancerous tissue (0.80 [0.64-0.85] vs. 0.58 [0.37-0.75]; p <0.001) (Figure 1B), with no difference in total 14,15-DHET level (including episomal and transformed DHET from 14,15-EET by sEH) (Figure 1C).

### Expression of enzymes for EET synthesis and degradation, and their association with clinicopathological variables in BC patients

**Figure 2:**
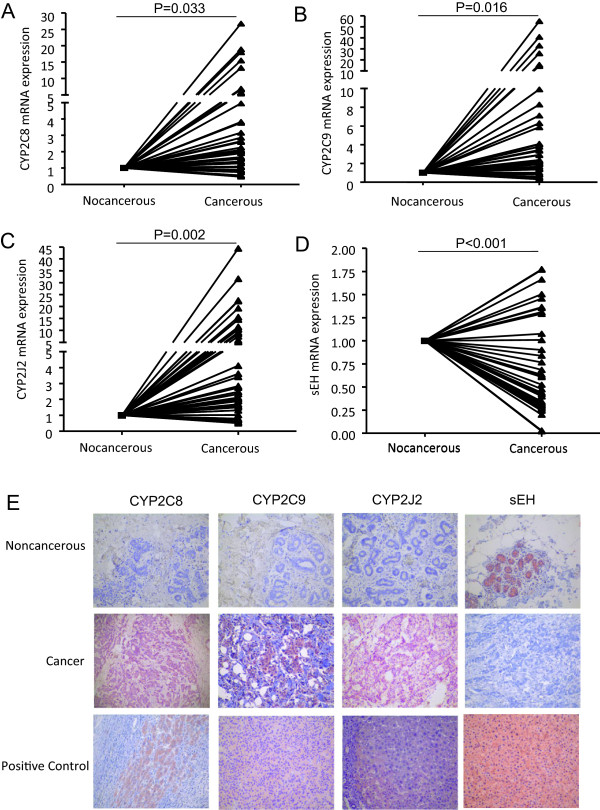
**Expression and distribution of CYP2C8, 2C9, and 2J2, and sEH in breast cancer.** qRT-PCR analysis of mRNA expression **(A-D)** and immunohistochemistry **(E)** of CYP2C8, 2C9, and 2J2, and sEH in breast cancer and paired adjacent noncancerous tissue. Human hepatocellular carcinoma tissues were used as a positive control. The red staining was by AEC and the nuclei were lightly stained with haematoxylin (original magnification 200X).

The mRNA levels of enzymes responsible for EET synthesis ranged from 4.4- to 7.5-fold greater BC than noncancerous tissue: CYP2C8 (4.40-fold), 2C9 (7.55-fold) and 2J2 (7.19-fold) (Figure 2). In contrast, sEH, responsible for EET degradation, was reduced (52.8% of normal) (Figure 2). Also, CYP2C9 protein expression was the most prevalent in the 40 cases of BC and adjacent noncancerous tissue, accounting for about 60% (24/40) and 45% (18/40), respectively, of the enzymes responsible for EET synthesis. Equal protein expression was observed for CYP2C8 and 2J2 in BC (30%) and noncancerous (10%) tissue (Table 1). The proportion of sEH protein expression was 40% (16/40) and 60% (24/40) in BC and adjacent noncancerous tissue, respectively. In addition, immunoreactivity was increased for CYP2C8 and 2J2 but not 2C9 and decreased for sEH in BC tissue (Table 1). These results indicate that, consistent with the elevated EET levels reported above, the enzymes responsible for EET synthesis are elevated, whereas the enzyme responsible for EET degradation is decreased in BC tissue.

**Table 1:** **EET-related gene expression in noncancerous tissue and adjacent breast cancer tissue**

EET-related genes	Non-cancerous tissue	Breast cancer tissue, no. of samples (%)			P value*
		-	+	++	
CYP2C8	-	28 (70%)	4 (10%)	4 (10%)	**0.019**
	+	0	0	2 (5%)	
	++	0	0	2 (5%)	
CYP2C9	-	12 (30%)	6 (15%)	4 (10%)	0.094
	+	4 (10%)	2 (5%)	6 (15%)	
	++	0	2 (5%)	4 (10%)	
CYP2J2	-	28 (70%)	6 (15%)	2 (5%)	**0.019**
	+	0	0	2 (5%)	
	++	0	0	2 (5%)	
sEH	-	10 (25%)	6 (15%)	0	**0.034**
	+	6 (15%)	2 (5%)	4 (10%)	
	++	8 (20%)	2 (5%)	2 (5%)	

Sum of frequency and intensity scores: 0 or 1, negative (−); 2 or 3, moderately positive (+); 4 to 6, highly positive (++).

*Spearman correlation was used to analyze the correlation of clinicopathological variables and EET-related genes expression.

The CYP2C8 protein level correlated with estrogen receptors (ER) and Ki67 status (P =0.011 and P =0.037, respectively); the CYP2C9 level correlated with Ki67 status (P =0.007); the CYP2J2 level correlated with histological grade and tumor size (P =0.036 and P =0.047, respectively); and sEH level was negatively correlated with tumor size, ER and Ki67 status (P =0.021; P =0.003 and P <0.001, respectively) (Table 2). No relationship was found with age, lymph-node metastasis, histological grade, C-erbB-2 and progesterone receptor (PR) expression.

**Table 2:** **Relationship of EET-related gene expression and clinical characteristics of BC patients**

Variable		CYP2C8			CYP2C9			CYP2J2			sEH		
		-	+	P value*	-	+	P value*	-	+	P value*	-	+	P value*
Age, years	≤50	12	4	0.573	8	8	0.292	12	4	0.573	12	10	0.618
	>50	16	8		8	16		16	8		12	6	
Histological grade	I	8	2	0.821	8	6	0.879	14	2	**0.036**	6	4	0.131
	II	12	6		6	8		10	4		8	10	
	III	8	4		2	10		4	6		10	2	
Tumor size, cm	≤2	8	0	0.058	6	3	0.089	9	1	**0.047**	2	7	**0.021**
	2-5	18	10		10	17		17	7		20	7	
	≥5	2	2		0	4		2	4		2	2	
Lymph node metastasis	Negative	14	4	0.332	12	12	0.114	19	5	0.166	10	6	0.792
	Positive	14	8		4	12		9	7		14	10	
ER status	-	6	8	**0.011**	4	10	0.279	8	6	0.193	4	10	**0.003**
	+	22	4		12	14		20	6		20	6	
Ki67 proportion	≤14%	8	8	**0.037**	2	14	**0.007**	10	6	0.49	4	12	**<0.001**
	>14%	20	4		14	10		18	6		20	4	
C-erb-B2 staining	−/+	22	6	0.13	14	14	0.079	24	8	0.221	20	10	0.159
	++/+++	6	6		2	10		4	4		4	6	
PR status	-	6	2	1	2	6	0.439	8	0	0.079	8	5	0.89
	+	22	10		14	18		20	12		16	11	

ER, estrogen receptors; PR, progesterone receptor.

*Spearman correlation was used to analyze the correlation of clinicopathological variables and EET-related genes expression.

### Effect of CYP450 and sEH on proliferation and migration of BC cells

**Figure 3:**
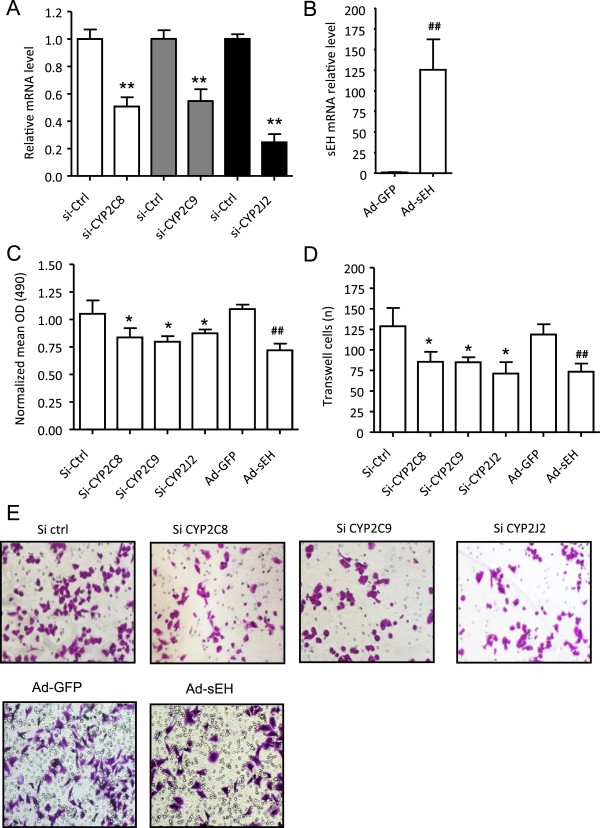
**Blockage of cancer-derived EETs by siRNA targeting CYP or overexpression of sEH inhibits proliferation and migration of breast cancer cells.** RT-PCR analysis of mRNA expression of CYP2C8, 2C9, and 2J2, and sEH in MDA-MB-231 cells treated with siRNA **(A)** or adenovirus-sEH **(B)** for 24 hr. **(C)** MTT assay of cell proliferation, expressed as the normalized mean OD_490_. **(D)** Transwell assay for cell migration. The number of migrated cells was measured by counting 5 randomly chosen fields under a microscope. **(E)** Representative transwell assay of cells stained with crystal violet (200X). Data are mean ± SD from 3 independent experiments each performed in triplicate (*P < 0.05; **P < 0.01 compared with Si-RNA control. ^##^P < 0.01 compared with Ad-GFP).

To further investigate the functional role of CYP2C8, 2C9, and 2J2 and sEH in BC cells, we transfected CYP2C8, 2C9 and 2J2 siRNA into MDA-MB-231 cells. At 24 h after transfection, mRNA expression was knocked down 57.9 ± 10.6%, 48.0 ± 4.5% and 63.5 ± 3.6%, and cell proliferation decreased by 24.5 ± 7.2%, 29.1 ± 5.0%, and 22.7 ± 3.5%, respectively (Figure 3C). Meanwhile, the proportion of migrating cells was also decreased by about 49.1 ± 10.2%, 44.9 ± 7.9%, and 50.9 ± 11.1%, respectively (Figure 3D, E). To overexpress sEH exogenously, we infected MDA-MB-231 cells with Ad-sEH. At 24 hr after infection, sEH mRNA was approximately 125.3-fold higher with Ad-sEH than Ad-GFP infection (Figure 3B). With Ad-sEH infection, cell proliferation was decreased about 31.4 ± 5.3% as compared with Ad-GFP transfection (Figure 3C) and the proportion of migrating cells was decreased about 45.8 ± 9.1%, (Figure 3E).

## Discussion

The level of EETs, as well as their synthetic and metabolic enzymes, has attracted great interest as potential therapeutic targets for renal disease, cardiovascular disease, inflammation and cancer [3, 6, 9, 21]. Indeed, previous in vivo and in vitro studies, including ours, have shown that CYP2C8, 2C9, and 2JC promote cancer cell proliferation, migration, angiogenesis, survival and invasion in several types of cancer such as hepatocellular carcinoma, esophageal carcinoma, and malignant hematologic disease [5, 6, 17]. However, the characteristics of EET expression and their synthetic and metabolic enzymes are not clear in BC. In the present study, we find a high level of 14,15-EET with increasing CYP2C8, 2C9 and 2J2 expression, and decreasing sEH mRNA and protein expression in BC as compared with adjacent noncancerous tissue. We further show that knockdown of CYP2C8, 2C9 and 2J2 or overexpression of sEH inhibits the proliferation and migration of BC cells.

CYP450s were thought to promote cancer progression primarily in the major metabolic organs, such as liver, kidney and epithelium. However, recent studies have detected a sustained high level of EETs by upregulated CYP2C8, 2C9 and 2J2, or downregulated sEH in various cancers such as renal, lung, basal cell, bladder, ovarian, colon, and prostate cell carcinomas [3, 7, 10, 22]. BC tissue shows a relatively high percentage and strong immunopositivity of CYP4X1, 2S1 and 2U among twenty-one P450 panels [13]. CYP3A4 is also a highly active arachidonic acid epoxygenase that promotes Stat3-mediated BC cell growth in part via (±)-14,15-EET biosynthesis [18]. Similarly, CYP2C is detected in 80% of BC tissue, based on a recent tissue microarray study [13].

We find that CYP2C9 and 2C8 are expressed in 60% and 30% of BC tissue, respectively. Both CYP2C8 and 2C9 protein levels positively correlate with Ki67 status, a biomarker for cell proliferation. The modest immunoreactive staining we find for CYP2J2 protein is consistent with previous studies [3, 10]. Despite the relatively low CYP2J2 prevalence, CYP2J2 protein expression is positively correlated with histological grade and tumor size, which is consistent with previous in vivo and in vitro studies [3, 10, 21]. Moreover, sEH expression is lower in BC than adjacent noncancerous tissue and negatively correlates with tumor size, ER and Ki67 expression. The elevated CYP2C8, 2C9, and 2J2 expression observed here may represent aggressiveness of BC cells and might predict worse cell behavior in BC patients.

Evidence from Panigrahy and colleagues has shown that elevated EET levels in endothelial cells leads to the development of tumor-associated angiogenesis and promotes metastasis [6, 23]. In vitro and in vivo studies also indicate that EETs may promote cancer progression by directly inducing cancer cell proliferation, survival, migration, and invasion, by changing the tumor microenvironment (inducing angiogenesis) and/or inducing immunosuppression in an autocrine and/or paracrine manner. This mechanism supports the role of CYP450s as potential tumor-promoting enzymes [3, 5]. For example, downregulated CYP2C expression and/or its enzymatic activity may provide a safe and effective strategy to treat non-small cell lung cancer [22]. Treatment of glioblastoma-bearing rats with CYP epoxygenase inhibitors attenuated tumor growth and tumor-associated angiogenesis [24]. Similarly, overexpression of sEH decreased HepG2 cell proliferation and induced cell cycle arrest [19]. Treating mice with a CYP2J peptide inhibited tumor growth by activating host antitumor immunity at an initial stage of an implanted murine bladder tumor [25]. In our study, consistent with the elevated 14,15-EET levels in BC tissue, elevated CYP2C8, 2C9 and 2J2 expression and reduced sEH expression might contribute to increased 14,15-EET levels and promote breast carcinogenesis. This result is further supported by our in vitro findings showing that knockdown of CYP2C8, 2C9 or 2J2 and overexpression of sEH inhibits the proliferation and slows the migration of BC cells.

Although the expression and role of CYP2C8, 2C9, and 2J2, and sEH have been reported in several tumors, their regulation is largely unknown. Evidence from our previous study showed that hypermethylation of the sEH promoter in HepG2 cells suppresses its transcription by an SP-1–dependent mechanism [19]. Human CYP2J2 and 2C8 are post-transcriptionally regulated by microRNAs let-7b and 103/107, respectively [26, 27]. EET receptor(s) have not yet been clearly identified, but the putative GPCR/PPAR/RXR pathway, endothelial growth factor receptor (EGFR) and vascular endothelial growth factor signaling are potential targets for EETs to promote tumor growth and metastasis [28–30]. Multiple pathways, including EGFR/PI3K/Akt, EGFR/mitogen-activated protein kinase, tumor necrosis factor-α and pro-metastatic matrix metalloproteinases are also involved in the mechanism by which EETs induce cancer cell proliferation and survival [3, 7, 21]. Thus, to further understand the molecular and biological mechanisms, and develop EET receptor antagonists as antitumor agents of EETs in malignant diseases, the EET receptor must be identified.

## Conclusion

In conclusion, increased EET levels in BC might be due to upregulation of CYP2C8, 2C9, and 2J2 and downregulation of sEH, and knockdown of CYP2C8, 2C9, and 2J2 and overexpression of sEH could partially attenuate the proliferation and migration of BC cells. These molecules might be novel therapeutic targets for the treatment of BC.

## Abbreviations

EET: Epoxyeicosatrienoic acid
sEH: Soluble epoxide hydrolase
CYP: Cytochrome P450
BC: Breast cancer
ER: Estrogen receptors
PR: Progesterone receptor
IHC: Immunohistochemical.
